# Recognition Memory and Prefrontal Cortex: Dissociating Recollection and Familiarity Processes Using rTMS

**DOI:** 10.1155/2008/568057

**Published:** 2008-04-11

**Authors:** Patrizia Turriziani, Massimiliano Oliveri, Silvia Salerno, Floriana Costanzo, Giacomo Koch, Carlo Caltagirone, Giovanni Augusto Carlesimo

**Affiliations:** ^1^Dipartimento di PsicologiaUniversità di PalermoPalermoItaly; ^2^IRCCS Fondazione Santa LuciaRomaItaly; ^3^Clinica NeurologicaUniversità di Roma Tor VergataRomaItaly

**Keywords:** Recognition memory, recollection, familiarity, prefrontal cortex

## Abstract

Recognition memory can be supported by both the assessment of the familiarity of an item and by the recollection of the context in which an item was encountered. The neural substrates of these memory processes are controversial. To address these issues we applied repetitive transcranial magnetic stimulation (rTMS) over the right and left dorsolateral prefrontal cortex (DLPFC) of healthy subjects performing a remember/know task. rTMS disrupted familiarity judgments when applied before encoding of stimuli over both right and left DLPFC. rTMS disrupted recollection when applied before encoding of stimuli over the right DLPFC. These findings suggest that the DLPFC plays a critical role in recognition memory based on familiarity as well as recollection.

## 1. Introduction

Recognition memory can be based on the recollection of specific information associated with a previous episode and on the assessment of the familiarity of an item [18]. However, it remains unclear whether these two forms of memory reflect distinct processes at a neural level. One view is that regions in the medial temporal lobe contribute to both familiarity and recollection and that recollection additionally depends on the prefrontal cortex [10,11]. Consistent with this idea, studies of patients with focal prefrontal lesions have reported disproportionate impairments on tests of recall and source memory that are thought to be sensitive to recollection [8,9].

An alternative view is that distinct medial temporal regions differentially contribute to familiarity and recollection, whereas the prefrontal cortex supports both these processes [1,18]. This idea draws support from some neuropsychological [4,5], neuroimaging [2,7,12, 20] and electrophysiological [3,6,16] studies in humans and from single-unit studies in monkeys [17] that link lateral prefrontal activity to both familiarity and recollection.

Another critical issue is whether recollection and familiarity can be best conceived as differing primarily in terms of retrieval processing, or whether they additionally differ at encoding. Some investigations suggest that recollection and familiarity rely on a similar neural system during encoding and may only be independent and dissociable at the time of retrieval, when controlled processing is essential for full recognition. Conversely, some evidence suggest that familiarity and recollection show distinct patterns during both encoding and retrieval processing [5,6].

ISSN 0953-4180/08/$17.00 © 2008 – IOS Press and the authors. All rights reserved

Table 1
Recognition judgments made to studied and new items at retrieval for each experimental condition. Data are given as means (SD)

| | Studied items | | New items | |
|---|---|---|---|---|
| **Experimental blocks** | Remember (%) | Know (%) | Remember (%) | Know (%) |
| Sham | 0.74 (0.14) | 0.17 (0.13) | 0.10 (0.09) | 0.12 (0.09) |
| R-Encoding short delay | 0.80 (0.12) | 0.16 (0.13) | 0.12 (0.33) | 0.23 (0.16) |
| R-Encoding long delay | 0.58 (0.29) | 0.26 (0.25) | 0.07 (0.18) | 0.10 (0.19) |
| L-Encoding short delay | 0.76 (0.15) | 0.20 (0.17) | 0.09 (0.21) | 0.23 (0.25) |
| L-Encoding long delay | 0.75 (0.18) | 0.15 (0.14) | 0.07 (0.14) | 0.14 (0.16) |
| R-Retrieval short delay | 0.80 (0.18) | 0.12 (0.19) | 0.12 (0.22) | 0.08 (0.13) |
| R-Retrieval long delay | 0.76 (0.19) | 0.14 (0.23) | 0.12 (0.27) | 0.14 (0.16) |
| L-Retrieval short delay | 0.68 (0.24) | 0.26 (0.25) | 0.07 (0.14) | 0.12 (0.13) |
| L-Retrieval long delay | 0.85 (0.14) | 0.07 (0.09) | 0.12 (0.24) | 0.09 (0.12) |

Following this theoretical framework, the aim of our study was to investigate: 1) the role of the DLPFC on recollection and familiarity; 2) whether recollection and familiarity are differently lateralised in the DLPFC; 3) whether recollection and familiarity may dissociate in terms of encoding and retrieval processing.

We studied the interfering effects of repetitive transcranial magnetic stimulation (rTMS) of the right and left DLPFC on recollection and familiarity processes during a recognition memory test of visually presented faces.

## 2. Material and methods

Sixteen healthy Italian right-handed subjects (4 men; 12 women; mean age: $23.4 \pm 3.1$ years) recruited from University of Rome Tor Vergata participated in the experiments. None of the subjects had any history of neurological or psychiatric illness. Written informed consent was obtained from all subjects prior to participating in the study, which had been approved by the institutional review board.

Visual stimuli consisted of two hundred and fifty six unknown faces. Sixteen stimuli were presented for six hundred ms in the encoding phase. During the retrieval phase, eight faces previously studied and eight non studied faces were presented. These stimuli were counterbalanced across participants so that each set served as old or new items at retrieval for different subjects. During the retrieval phase, subjects made "remember," "know," or "new" judgments [15]. Instructions for the test phase included a description of the appropriate use of the remember, know, and new categories. Subjects were instructed to respond "remember" if they were certain they had seen the face and could recollect specific associations that occurred at study, "know" if they were certain about previously studying the face and could not recollect any specific associations, and "new" if they were certain they had never previously studied the face. This allowed us to verify that remember and know responses were associated with recollection and familiarity processes, respectively [18]. Behavioural responses were collected during ten blocks of study-test trials. There was a 5 min delay between the study phase and the test phase.

rTMS was delivered in trains of 6 stimuli at 20 Hz frequency over F3 and F4 scalp sites of the 10-20 EEG system. rTMS was delivered with an event related design, during presentation of stimuli. For both encoding and retrieval phases, rTMS was applied at two different intervals after the onset of the visual stimulus: from 0 to 300 ms and from 300 to 600 ms. These intervals were selected according to the findings of previous event related potential studies of familiarity and recollection processes [6]. The same intensity and timing of rTMS were used for sham stimulation of each of the test locations (F3 and F4), in which the coil was held close to the target site, but angled away so that no current was induced in the brain. Sham conditions were randomly intermingled with test conditions. The order of the different experimental conditions was randomised across subjects. Responses were measured in terms of accuracy (mean number of correct responses).

## 3. Results

Table 1 shows the mean proportions of R and K judgments to studied items and new items for each experimental condition. Results from the remember–know recognition test were used to estimate indices of recollection and familiarity. To correct for the underestimation of familiarity inherent in the remember/know design, we derived the estimates of recollection and familiarity from the data using the formulae provided

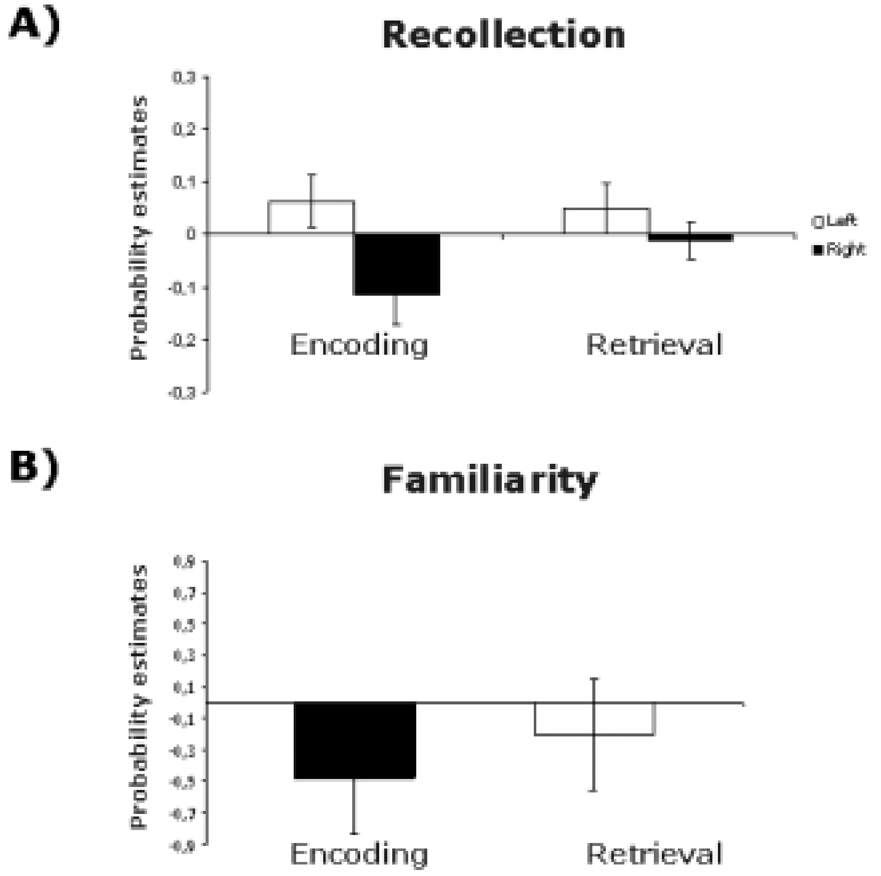

Fig. 1. **A**) Estimates of recollection as a function of side (i.e. left vs. right hemisphere) and phase of stimulation (i.e. encoding vs. retrieval). Recollection is selectively disrupted when rTMS was applied over the right PFC during the encoding phase. Error bars depict the standard error of the mean across subjects; **B**) Estimates of familiarity as a function of phase of stimulation (i.e. encoding vs. retrieval). Familiarity is disrupted when rTMS pulses are applied during the encoding process. Error bars depict the standard error of the mean across subjects.

by Yonelinas and Jacoby [19]. For this reason, we conducted two distinct statistical analyses for recollection and familiarity data. Normalized data (real-sham rTMS) were used for the analysis.

Statistical analysis was performed using ANOVA for repeated measures with Hemisphere (right vs. left) and Phase (encoding vs. retrieval) and Delay (short vs. long) as within-subjects factors.

As to the recollection, ANOVA revealed significant main effect of Hemisphere [F (1,15) = 4.97; $p > 0.05$]: disruption of recollection is selectively present when rTMS is applied over the right PFC. There was also a significant effect of Phase of Stimulation [F (1,15) = 6.5; $p < 0.05$]: disruption of recollection is selectively present when rTMS is applied during the encoding phase. There was also significant Hemisphere x Phase of stimulation interaction [F (1,15) = 5.14; $p < 0.05$]: disruption of recollection is selectively present when rTMS is applied over the right PFC during the encoding phase (Fig. 1a). Finally, there was a significant Hemisphere x Delay interaction [F (1,15) = 6.41; $p < 0.05$]: rTMS of the right PFC disrupts recollection only if applied at long delays from visual stimulus presentation.

As to the familiarity, the ANOVA did not reveal any significant Hemisphere effect [F (1,15) = 3.8; $p > 0.05$]. In fact, familiarity is reduced in the same way when rTMS is applied on both the left and the right hemisphere. These findings suggest that familiarity is widely dependent on the activity of both the left and the right DLPFC. A significant main effect of Phase of stimulation was found [F (1,15) = 7.5; $p < 0.05$]. The amount of familiarity is significant lower when rTMS pulses are applied during the encoding phase (Fig. 1b). Moreover, there was a significant Delay effect [F (1,15) = 34.3; $p < 0.05$]. The familiarity is lower when rTMS is applied during the second part of stimulus presentation.

## 4. Discussion

In the present study estimates of familiarity and recollection derived from the remember/know procedure suggest that these processes are specifically disrupted when rTMS trains are applied on different experimental conditions.

The adopted rTMS procedure, with high-frequency trains applied on-line (i.e. during task execution) allows transiently interfering with the ongoing cognitive task, by adding random noise activity in the stimulated region. On the other hand, slower rTMS frequencies (i.e. 1 Hz trains) are mostly applied with an off-line design (i.e. before the execution of the cognitive task) and induce a long-term modulation of excitability of the stimulated region. Therefore, this procedure presents a lower temporal resolution and is less suitable for exploring the chronometry of the cognitive effects, which was one of the aims of the present study.

The main findings regarding recollection show that the remember judgment is disrupted when rTMS is applied on the right hemisphere and in particular at long delay. In addition, there is a decline of recollection when rTMS is applied during the encoding processing. These findings suggest that the encoding process is strictly related to the amount of subsequent recollection. It can be hypothesized that the decline of recollection is due to the inability to create strong associations between the item and the context during the encoding process.

The main findings regarding familiarity show that the familiarity judgment is disrupted when rTMS is applied on both the left and the right hemisphere, suggesting that familiarity is bilaterally represented. The decline of familiarity is more robust when rTMS is applied during the encoding process and when the trains of TMS pulses are delivered at long delay following the onset of stimulus presentation.

These findings extend the results of previous rTMS studies on the role of prefrontal cortex in episodic memory retrieval [13,14]. They suggest that the DLPFC is a structure necessary for both familiarity and recollection processes. The results additionally suggest a relative dissociation between the contribution from left and right PFC to recollection and familiarity processes. In particular, the right DLPFC is critical for recollection, while both right and left DLPFCs are critical for familiarity. Finally, this study suggests that encoding rather than retrieval phases are critical for both familiarity-based and recollection-based memory recognition.
